# Pseudomonal sternal abscess and osteomyelitis as an initial manifestation of common variable immunodeficiency

**DOI:** 10.1128/asmcr.00014-26

**Published:** 2026-05-29

**Authors:** Marina Makram, Elie Bou Sanayeh, Hadi Itani, Elie Moussa, Ahmed Zayed, Allison Glaser

**Affiliations:** 1 Department of Internal Medicine, Staten Island University Hospital, Northwell Health 7601 , New York, New York, USA; 2 Department of Infectious Diseases, Staten Island University Hospital, Northwell Health 7601 , New York, New York, USA; Rush University Medical Center, Chicago, Illinois, USA

**Keywords:** common variable immunodeficiency, hypogammaglobulinemia, sternal abscess, osteomyelitis, *Pseudomonas aeruginosa*

## Abstract

**Background:**

Common variable immunodeficiency (CVID) is the most prevalent symptomatic primary immunodeficiency in adults and is characterized by hypogammaglobulinemia and recurrent bacterial infections. The initial presentation of sternal osteomyelitis and abscesses, particularly due to *Pseudomonas aeruginosa*, is exceedingly rare.

**Case Summary:**

We report the case of a 39-year-old, previously healthy male who presented with progressive right-sided chest pain and an anterior chest wall mass. Imaging revealed sternal bone erosion with an associated parasternal soft-tissue collection. Surgical exploration revealed two sternal abscess cavities, and cultures grew pan-susceptible *Pseudomonas aeruginosa*. Laboratory studies revealed profound hypogammaglobulinemia, and flow cytometry revealed a small monoclonal B-cell population. Autoimmune and infectious workup results were unremarkable. The patient underwent serial surgical debridement and received 6 weeks of intravenous cefepime with initiation of intravenous immunoglobulin therapy for the newly diagnosed CVID. At the 3-month follow-up, there was complete clinical resolution with no evidence of recurrent infection.

**Conclusion:**

This case highlights a rare presentation of CVID with sternal osteomyelitis and abscess caused by *Pseudomonas aeruginosa*. This underscores the importance of considering primary immunodeficiency in adults with atypical or severe infections and highlights the diagnostic and therapeutic challenges posed by such unusual presentations. Early identification and immunoglobulin replacement therapy are critical for achieving favorable outcomes.

## INTRODUCTION

Common variable immunodeficiency (CVID) is a heterogeneous primary humoral immunodeficiency characterized by low levels of immunoglobulins (IgG, IgA, and often IgM), resulting in increased susceptibility to infections and a broad spectrum of non-infectious complications (1, 2). CVID is the most frequently diagnosed symptomatic primary immunodeficiency in adults, with an estimated prevalence of approximately 1 in 25,000 individuals (3, 4). The underlying pathogenesis involves defects in B-cell function and impaired antibody production, compromising the humoral immune response (5). Diagnosis is based on persistently low serum IgG levels, often accompanied by decreased IgA and/or IgM levels, a poor antibody response to vaccines, and exclusion of secondary causes of hypogammaglobulinemia. The American Academy of Allergy, Asthma, and Immunology recommends considering CVID in patients older than 4 years with these laboratory findings (6). Most cases are recognized between the ages of 20 and 50 years, but diagnosis can occur at any age (4).

The clinical presentation is highly variable, but recurrent respiratory tract infections, such as pneumonia, chronic bronchitis, and sinusitis, are the most common initial manifestations (1, 2). The most frequently encountered pathogens are encapsulated bacteria, notably *Streptococcus pneumoniae*, *Haemophilus influenzae*, and *Neisseria* species. *Mycoplasma* species are also observed (6, 7). In addition to infections, patients may develop autoimmune cytopenia (e.g., immune thrombocytopenia and hemolytic anemia), chronic lung disease (including bronchiectasis and interstitial lung disease), gastrointestinal inflammation, lymphoproliferative disorders (lymphadenopathy and splenomegaly), granulomatous disease, and an increased risk of certain malignancies, particularly lymphoma (1, 2, 4, 7). While severe bacterial infections, such as sepsis and meningitis, are reported in a minority of cases, organ or tissue abscesses are exceedingly rare as a presenting feature of CVID (1, 2, 7). In extensive cohort studies and systematic reviews, abscess formation, especially in deep organs or the mediastinum, has not been described as a typical manifestation (1, 2, 7, 8).

Here, we describe the first case of a patient presenting with a mediastinal abscess and osteomyelitis secondary to *Pseudomonas aeruginosa*, who was subsequently diagnosed with CVID, highlighting an atypical and previously unreported presentation of this immunodeficiency.

## CASE PRESENTATION

A 39-year-old male with no significant medical history presented with right-sided chest pain and a palpable anterior chest wall mass, which developed 1 month after an episode of influenza and bronchitis treated with corticosteroids. The initial pain improved transiently with non-steroidal anti-inflammatory drugs but subsequently recurred and progressively worsened, prompting an emergency medical evaluation. He denied having a fever, cough, dyspnea, or a history of trauma. He never had a history of recurrent upper and lower respiratory infections and was never diagnosed with bronchiectasis before. He did not undergo any surgery in the past requiring sternotomy. He denied the use of illicit intravenous drugs.

Initial laboratory evaluation was largely unremarkable, with a white blood cell count of 10 × 10^3^/µL (reference range: 4.8–10.8 × 10^3^/µL). Inflammatory markers were elevated, including an erythrocyte sedimentation rate of 35 mm/h (reference range: 0–10 mm/h) and a C-reactive protein level of 49 mg/L (reference: <5 mg/L). Two sets of blood cultures were negative, and transthoracic echocardiography revealed no vegetation. Computed tomography of the chest (Fig. 1) demonstrated focal erosion or lucency of the right lower sternum with associated parasternal soft tissue swelling and subcutaneous fat stranding. Findings were suspicious for sternal osteomyelitis with associated soft-tissue phlegmon. No discrete abscess was identified. No pulmonary or airway pathologies were noted. Magnetic resonance imaging of the chest (Fig. 1) confirmed osseous involvement of the lower sternal body with an enhancing 4.2 × 3.2 × 3.7 cm infiltrative soft tissue mass centered at the costochondral junction. Findings were suspicious for soft-tissue phlegmon; however, an underlying neoplasm could not be excluded at this stage.

**Fig 1:**
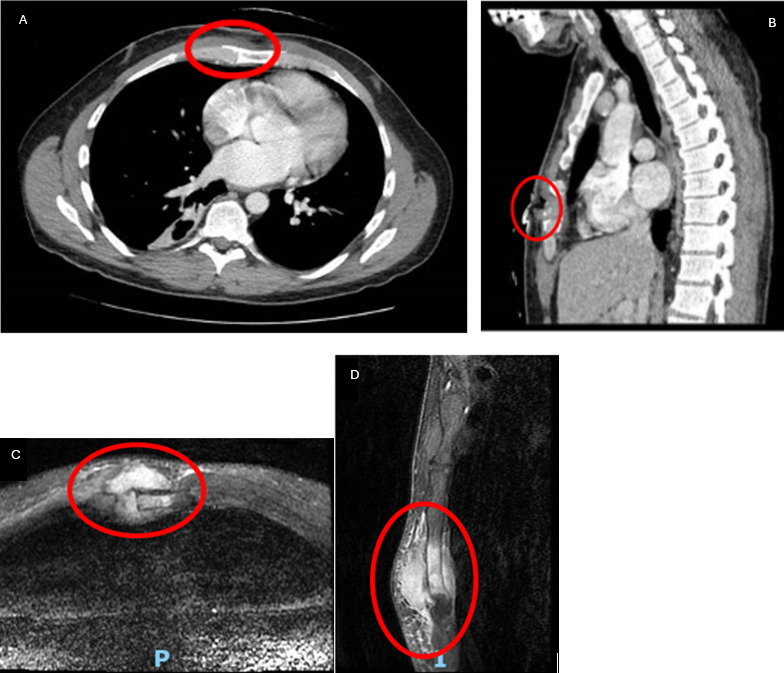
Computed tomography of the chest shows bone erosion and lucency of the lower right sternum with associated parasternal soft-tissue swelling and subcutaneous fat stranding (Panel **A**, axial view; Panel **B**, sagittal view). Magnetic resonance imaging of the chest (Panel **C**, axial view; Panel **D**, sagittal view) demonstrates a 4.2 × 3.2 × 3.7 cm enhancing infiltrative soft-tissue mass centered at the right lower anterior costochondral junction, with osseous involvement of the adjacent lower sternal body.

Surgical exploration revealed two distinct abscess cavities, which were drained, and wound vacuum-assisted closure therapy was initiated. Intraoperative wound cultures from multiple specimens showed pan-susceptible *Pseudomonas aeruginosa* (susceptible to cefepime, ceftazidime, ciprofloxacin, piperacillin-tazobactam, and other antipseudomonal agents). Histopathological examination of intraoperative tissue samples revealed fibroadipose and muscular tissues with acute and chronic inflammation and granulation tissue, consistent with abscess formation and no evidence of malignancy. The patient underwent serial debridement and washout followed by closure and drain placement. Blood culture results remained persistently negative during hospitalization.

Remarkably, serum immunoglobulin testing revealed marked hypogammaglobulinemia: IgG 106 mg/dL (reference range: 610–1,660 mg/dL), IgA <2 mg/dL (reference range: 84−499 mg/dL), and IgM 24 mg/dL (reference range: 35–242 mg/dL), confirmed on repeat testing. Flow cytometry revealed a small population of CD5+/CD23+ monotypic B cells, suggestive of monoclonal B-cell lymphocytosis or early chronic lymphocytic leukemia (CLL). A comprehensive autoimmune and infectious workup, including HIV testing (detection of HIV-1 p24 antigen and HIV-1/HIV-2 antibodies) and tuberculosis testing (wound acid-fast bacilli stain and culture, serum interferon-gamma release assay), was negative. To note that testing for other immunodeficiencies (e.g., chronic granulomatous disease) was not performed.

Based on clinical, radiographic, microbiological, and immunological findings, a diagnosis of *Pseudomonas* sternal abscess and osteomyelitis secondary to presumptive underlying common variable immunodeficiency (CVID) was established. The patient received a 6-week course of intravenous cefepime (2 g every 8 h) via a peripherally inserted central catheter (PICC) line and was referred to immunology and hematology for further management. He had a repeat immunoglobulin panel 1 week following discharge from the hospital, which showed persistently low immunoglobulin levels (particularly hypogammaglobulinemia). He was subsequently diagnosed with CVID, and intravenous immunoglobulin (IVIG) therapy was initiated. At the 3-month follow-up, the patient reported complete resolution of chest pain, had no evidence of recurrent infection, showed improved inflammatory marker levels, and remained clinically stable on maintenance IVIG therapy. Unfortunately, vaccine response testing was not performed, and CLL could not be ruled out due to changes in institutions during subsequent follow-ups.

## DISCUSSION

This case illustrates the intersection between atypical infection and the underlying immune dysfunction. *Pseudomonas* sternal abscess and osteomyelitis are uncommon in immunocompetent adults and are typically associated with risk factors, such as intravenous drug use, prior surgery involving the sternum, trauma, or immunosuppression. In this patient, the absence of these traditional risk factors, coupled with profound hypogammaglobulinemia, raised suspicion of an underlying immunodeficiency, ultimately leading to the diagnosis of CVID.

While respiratory tract infections, particularly those caused by encapsulated organisms, remain the most common initial presentation of CVID, severe bacterial infections, such as osteomyelitis, occur in up to 4% of patients, underscoring the susceptibility of this population to deep-seated and opportunistic infections (1). Osteoarticular involvement in CVID has become increasingly recognized, encompassing both infectious and non-infectious etiologies. Reported risk factors include low body weight and hepatomegaly, with the cumulative incidence of infectious osteoarticular complications approaching 17%. Autoimmune and degenerative mechanisms may contribute to the overall morbidity observed in these patients (9). Cutaneous manifestations are also frequent in CVID, with skin infections documented in approximately 40% of the patients. Among these, abscesses represent the most common cutaneous infectious complication, occurring in over 20% of affected individuals (8). Other presentations, such as cellulitis and furunculosis, may serve as sentinel events leading to the initial diagnosis of CVID, as described in several case reports (10). The spectrum of skin involvement extends beyond infectious lesions, encompassing atopic dermatitis and autoimmune dermatoses, which collectively reflect the underlying immune dysregulation characteristics of CVID (8, 10).

To our knowledge, this is the first reported case of CVID presenting with chest wall abscess and sternal osteomyelitis. Similar findings have been reported previously. Hatab et al. (11) reported a case of a patient with known CVID who developed aseptic abscesses of the psoas and spleen, characterized by the absence of infectious agents or positive cultures. Similar to our case, Stahl et al. (12) described a case in which a diagnosis of CVID was established following the discovery of a gangrenous peri-appendiceal abscess. These cases, together with ours, highlight the variable and sometimes atypical infectious presentation of CVID.

In our patient, management included surgical drainage of the abscess, followed by intravenous antibiotic therapy tailored to the culture sensitivities obtained during the operative washout. The isolate was susceptible to cefepime, and the patient exhibited an appropriate clinical response. Consequently, a 6-week course of intravenous cefepime was administered via a PICC line, resulting in the resolution of the infection. Management of CVID centers with lifelong immunoglobulin replacement therapy is effective in preventing infections and reducing disease-related morbidity. Immunoglobulin may be administered intravenously or subcutaneously with individualized dosing to achieve infection-free intervals rather than targeting a fixed IgG trough level. Recall that our patient had immunoglobulin panel result that reflected abnormally low IgG levels. This diagnostically relevant finding can often serve as a screening tool for the diagnosis of CVID (13). Ongoing monitoring is required for infectious and non-infectious complications, including autoimmune diseases, granulomatous inflammation, and malignancy. Adjunctive therapies may be necessary for the treatment of autoimmune or inflammatory manifestations. In contrast, chronic organ complications such as those affecting the lungs or gastrointestinal tract should be managed on a patient-specific basis. Finally, early and aggressive treatment of infections, including those caused by atypical pathogens, remains critical for optimizing the outcomes of CVID (5, 6, 14). Unfortunately, diagnostic delays remain common owing to the heterogeneous and multisystem nature of the disease, potentially resulting in irreversible organ damage if not recognized early (8).

The clinical implications of this case are twofold. First, it underscores the critical need for clinicians to maintain a high index of suspicion for an underlying immunodeficiency, including CVID, in adults presenting with atypical or severe infections such as sternal osteomyelitis, particularly in the absence of traditional predisposing factors. Second, early recognition and diagnosis of CVID are essential to enable prompt initiation of therapy, reduce morbidity, and prevent long-term complications, such as bronchiectasis, chronic organ dysfunction, and malignancy. Because CVID can initially present to multiple specialties, including dermatology, rheumatology, gastroenterology, and infectious diseases, broad multidisciplinary awareness is vital for timely detection and management (2, 5, 8, 14).

### Conclusion

This case underscores the importance of considering primary immunodeficiency disorders, such as CVID, in adult patients presenting with atypical or severe bacterial infections, even in the absence of a significant prior infectious history. This case highlights the need for multidisciplinary collaboration in diagnosing and managing unusual infections, as well as the broader implications of identifying underlying immunodeficiency syndromes in adult patients.
